# Transposable element insertion: a hidden major source of domesticated phenotypic variation in *Brassica rapa*

**DOI:** 10.1111/pbi.13807

**Published:** 2022-03-18

**Authors:** Xu Cai, Runmao Lin, Jianli Liang, Graham J. King, Jian Wu, Xiaowu Wang

**Affiliations:** ^1^ Institute of Vegetables and Flowers Chinese Academy of Agricultural Sciences Beijing China; ^2^ Southern Cross Plant Science Southern Cross University Lismore NSW Australia

**Keywords:** *Brassica rapa*, crop domestication, intraspecific diversification, pan‐genome, transposable element insertion

## Abstract

Transposable element (TE) is prevalent in plant genomes. However, studies on their impact on phenotypic evolution in crop plants are relatively rare, because systematically identifying TE insertions within a species has been a challenge. Here, we present a novel approach for uncovering TE insertion polymorphisms (TIPs) using pan‐genome analysis combined with population‐scale resequencing, and we adopt this pipeline to retrieve TIPs in a *Brassica rapa* germplasm collection. We found that 23% of genes within the reference Chiifu‐401‐42 genome harbored TIPs. TIPs tended to have large transcriptional effects, including modifying gene expression levels and altering gene structure by introducing new introns. Among 524 diverse accessions, TIPs broadly influenced genes related to traits and acted a crucial role in the domestication of *B. rapa* morphotypes. As examples, four specific TIP‐containing genes were found to be candidates that potentially involved in various climatic conditions, promoting the formation of diverse vegetable crops in *B. rapa*. Our work reveals the hitherto hidden TIPs implicated in agronomic traits and highlights their widespread utility in studies of crop domestication.

## Introduction

Transposable elements (TEs) are ubiquitous components of plant genomes (Feschotte *et al*., 2002). They have contributed to rapid adaptation and the domestication of crops through generation of novel traits (Bourque *et al*., 2018; Catlin and Josephs, 2021; Chuong *et al*., 2017; Niu *et al*., 2019; Quadrana *et al*., 2019; Sultana *et al*., 2017; Zhang *et al*., 2015). As early as 1948, Barbara McClintock observed that transposons were associated with color variegation in maize kernels and leaves (Mc, 1951), revealing the crucial role of TE in phenotypic variation. TE insertions, that are present in some individuals but absent in others are denoted as TE insertion polymorphisms (TIPs), and represent a major source of intraspecific variation. Specifically, TIPs are an important type of structural variation. In pan‐genomic studies of soybean and *B. rapa*, structural variations (SVs) have tended to be enriched within repetitive sequences (Cai *et al*., 2021; Liu *et al*., 2020b). Moreover, the TE insertions are usually nonrandom, many TEs are highly enriched in promoters and downstream regions of genes (Niu *et al*., 2019). TE insertions can lead to epigenetic modification, alter gene structure, and rewrite gene expression networks (Chuong *et al*., 2017; Dominguez *et al*., 2020; Sultana *et al*., 2017; Woodhouse *et al*., 2014).

Traditional crop domestication analysis has been limited to single reference genotypes and small mutations (SNPs and InDels). This largely masks the impacts of the vast majority of SVs on crop domestication. The rapid increase in available whole‐genome assemblies has moved plant genetics into the pan‐genomic era. The construction of a species’ pan‐genome enables us to resolve hidden genomic complexity involving SVs, and a re‐evaluation of their role in genome evolution and generation of phenotypic variation (Bayer *et al*., 2020). A large body of work has revealed that SVs have played crucial roles in specific plant adaptations and agronomic traits, including regulation of flowering time, fruit flavor, fruit size, and productivity (Alonge *et al*., 2020; Hufford *et al*., 2021; Liu *et al*., 2020b; Qin *et al*., 2021; Song *et al*., 2020). However, as a major class of SVs, TIPs are a poorly documented source of phenotypic variation (Dominguez *et al*., 2020).

The cruciferous species *B. rapa* has high economic importance, with distinct morphotypes widely cultivated as vegetables, fodder, condiment, and oilseed crops throughout the world. During domestication over a long period in Europe as well as in Asia, the species has provided a rich source of phenotypic variants responding to various climatic conditions (Warwick, 2011). For example, turnips that form enlarged roots are biennial and require vernalization to flower (Zhang *et al*., 2014); the *B. rapa* oilseed crops that are used for oil extraction establishes annual spring and biennial winter types (McAlvay *et al*., 2021); Chinese cabbages that form leafy heads vary in annual/biennial habit, presenting spring, summer, and autumn ecotypes (Su *et al*., 2018); and one East Asian vegetable crop caixin that is selected from pak choi (Cheng *et al*., 2016) bolts rapidly to form long and tender floral stems (Cheng *et al*., 2014; Zhao *et al*., 2005). These diverse morphological configurations represent important agronomic traits in *B. rapa*. Based on resequencing 199 diverse *B. rapa* accessions, Cheng *et al*. (2016) confirmed that leafy head and enlarged root traits were under strong selection during domestication and breeding (Cheng *et al*., 2016). Recently, based on pan‐genome analysis, we found that nearly 50% of SVs were enriched in repetitive sequences, while also being tightly associated with the domestication of distinct morphotypes (Cai *et al*., 2021). These findings hinted that TIPs may have played an important role in the domestication of *B. rapa* morphotypes.

Here, we present a novel approach for detecting TIPs in population‐scale based on the *B. rapa* pan‐genome and resequencing of 524 diverse genomes. Using this pipeline, we systematically identified *B. rapa* TIPs that had not previously been documented. We found that TE insertions have led to large transcriptomic changes, both altering gene expression and generating novel introns. Moreover, we found TIPs in TIP‐containing genes showing evidence of stronger selection pressure than nonsynonymous single nucleotide polymorphisms (SNPs). As examples, four TIP‐containing genes were further investigated as candidates potentially involved in climate change adaptation, promoting the formation of diverse *B. rapa* crops in various climatic condition.

## Results

### Transposable element content varies considerably in different *B. rapa* genomes

Transposable elements are major components of plant chromosomes, and play an important role in genome evolution (Chuong *et al*., 2017; Sultana *et al*., 2017). To investigate TE dynamics in different *B. rapa* genomes, we calculated TE content in 20 diverse *B. rapa* genomes. Eighteen accessions were employed for constructing the *B. rapa* pan‐genome in our previous study (Cai *et al*., 2021); the remaining two pak choi accessions (NHCC001 and ZYCX) were used in recently reported studies (Li *et al*., 2020, 2021), renamed as PCC and PCD in this study. Here, we expanded the representation of the *B. rapa* pan‐genome to include 20 genomes having a distinctive range of morphotypes (Figure S1).

**Figure 1:**
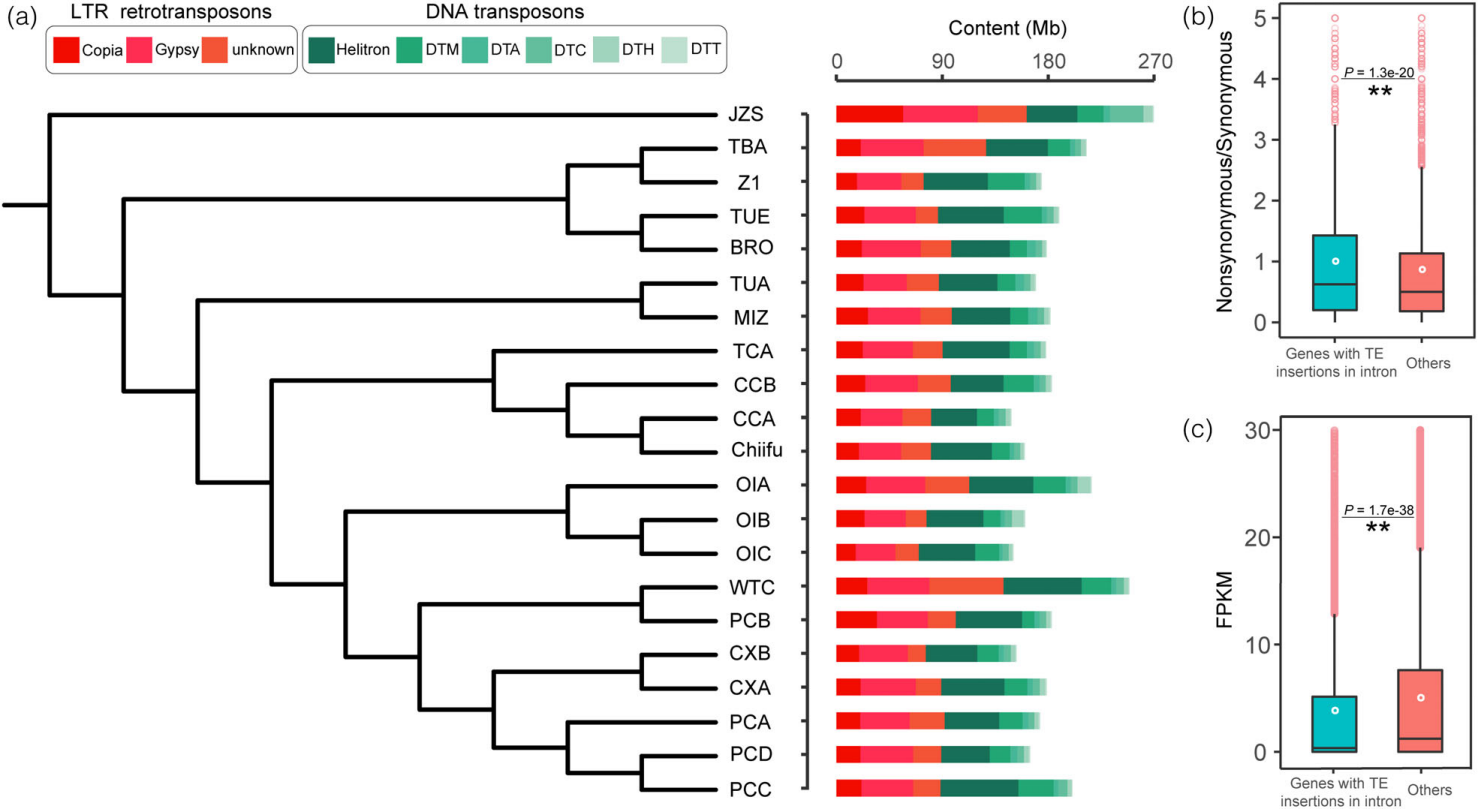
Characteristics of transposable elements (TEs) in *B. rapa* genomes. (a), The TE content in each of the 20 *B. rapa* genomes. The phylogenetic tree was constructed using single‐copy genes within the 20 *B. rapa* genomes, and the accession “JZS” (*Brassica oleracea*) was used as an outgroup. DNA transposons were classified into Helitron and other five major superfamilies (Feschotte and Pritham, 2007) DTM (Mutator), DTA (hAT), DTC (CACTA), DTH (PIF/Harbinger), and DTT (Tc1/Mariner). (b) The ratio of nonsynonymous SNPs to synonymous SNPs in genes with and without TE insertions in introns. (c) Relative transcription levels of genes with and without TE insertions in introns. Multiple comparisons were performed using the Student‐Newman–Keuls test with *a* = 0.01 (the same as presented in Figures 4 and 6).

Approximately 43.4%−53.5% of assembly sequences were annotated as TEs in the 20 *B. rapa* genomes (Table S1). The total TE content in each genome was positively correlated with the corresponding assembled genome size (*R* = 0.99, *P* = 3.8e–16) (Figure S2). The TE total sequence nucleotide counts in the WTC genome was 1.42 times that found in the Z1 genome. Furthermore, we calculated the relative contents of long terminal repeat retrotransposons (LTR‐RTs) and DNA transposons, the two predominant groups of plant TEs. Among the 20 genomes, the LTR nucleotide count varied two‐fold from 70.1 to 142.1 Mb, and mainly accounted for the differences in TE content among *B. rapa* genomes (Figure 1a). As the most abundant LTR, sequence length of LTR/Gypsy repeat elements was 33.7–53.4 Mb, accounting for 9.3%–13.2% of entire genomic sequences. For the second most abundant LTR, the average sequence length of LTR/Copia repeat elements was around half the length of the LTR/Gypsy, again varying ~two‐fold, from 16.6 to 34.6 Mb in the 20 *B. rapa* genomes. DNA transposons, comprised a total of 25.3–49.6 Mb, accounting for 6.5%–12.3% of the genomic sequences.

Transposable element insertions were closely associated with gene evolution. For the *B. rapa* reference genome (Chiifu‐401‐42, hereafter referred as Chiifu), a total of 9586 gene models harbored TE insertions within introns. We observed that the average ratio of nonsynonymous SNPs to synonymous SNPs was significantly higher in genes with TE insertions than those without (Figure 1b; *P* = 1.3e–20), suggesting that TE insertions might be associated with the higher rate of nonsynonymous mutations. Meanwhile, we found that the average expression level of genes with TE insertions within introns was significantly lower than that of the genes without TE insertions (Figure 1c; *P* = 1.7e–38). Although these findings have suggested a link between TE insertions and the dynamics of genome size and gene evolution, to date there has been no systematic study of the effect of TE insertions on the domestication of *B. rapa* morphotypes.

### A pipeline to retrieve population‐scale TE insertion polymorphisms

**Figure 2:**
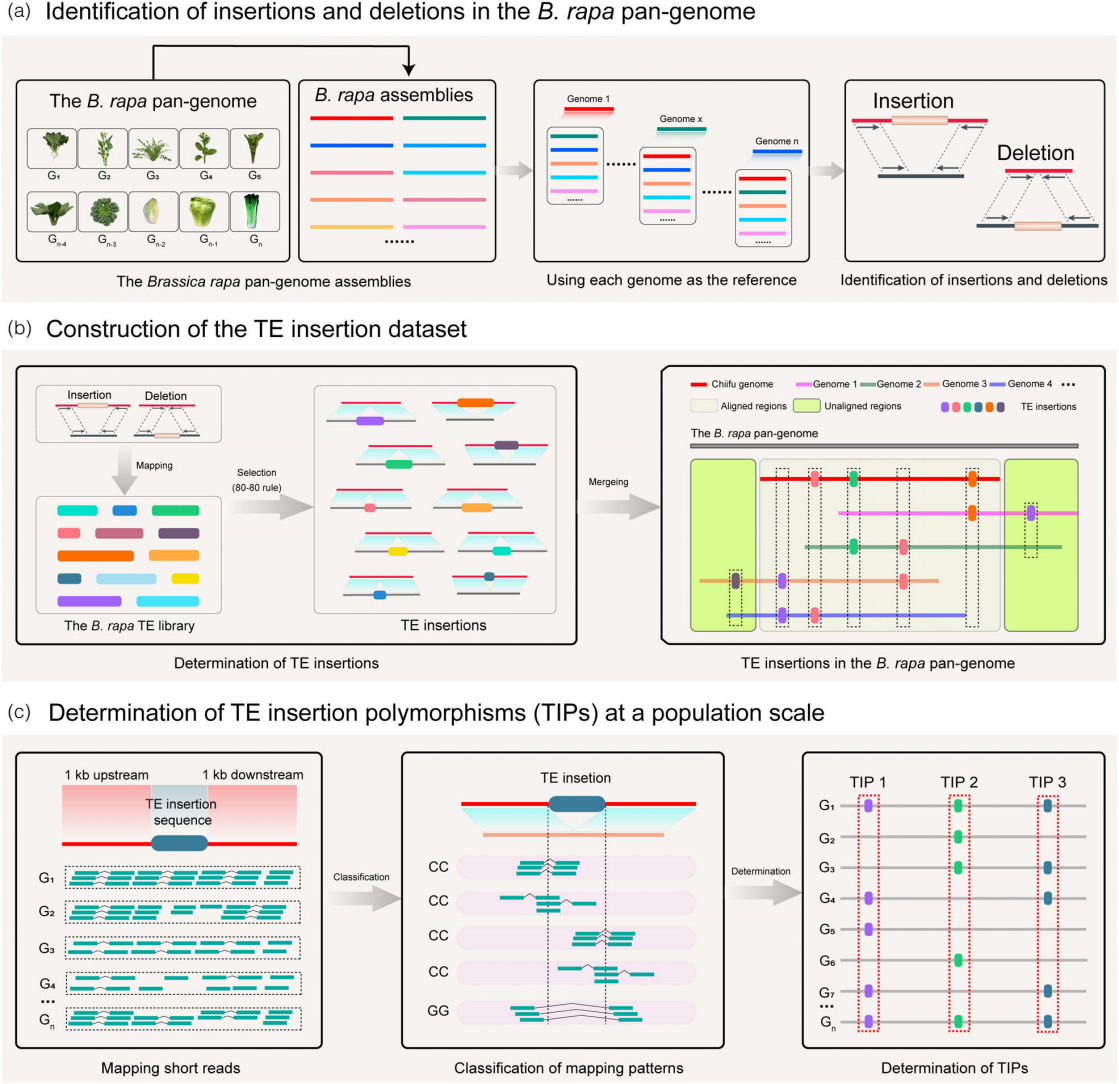
The pipeline for identifying transposable element (TE) insertion polymorphisms (TIPs) in *B. rapa* genomes by a combination of pan‐genomic and population resequencing strategies. (a) Identification of insertions and deletions in the *B. rapa* pan‐genome. Each of the 20 *B. rapa* genomes is used as the reference for comprehensively identifying insertions and deletions. (b) Construction of the TE insertion dataset. Each insertion or deletion sequence was mapped onto the *B. rapa* TE library to determine TE insertions in *B. rapa*. TE insertions in the pan‐genome were divided into TE insertions in the “aligned regions” and “unaligned regions.” (c) Determination of TIPs in 524 *B. rapa* genomes. Short reads were mapped to the TE sequence and flanking sequences for genotyping. Based on the mapping patterns, we genotyped each TE insertion and calculated polymorphic TE insertions. “G_1,_” “G_2,_”‡, “G_n_” represent each re‐sequenced *B. rapa* genome. “CC” and “GG” indicate the two genotypes with and without TE insertion, respectively, in the corresponding resequenced genome.

Analysis of TIPs in population scale had been difficult due to limited number of *de novo* assembled genome for a specific species. To dissect the role of TIPs played in genome evolution, we developed a pipeline to identify population‐scale TIPs based on a pan‐genome analysis and large‐scale resequencing of *B. rapa* accessions. The pipeline for identifying population‐scale TIPs employed three sequential steps: identification of insertions and deletions (Figure 2a), construction of the TE insertion dataset (Figure 2b), and determination of TIPs at a population scale (Figure 2c) (Appendix S1). First, pairwise comparison of the 20 *B. rapa* genomes enabled retrieval of TE insertions, that then were collated to represent the complete insertion dataset. Second, each TE insertion from this dataset was further compared against each of the 524 *B. rapa* short‐read resequenced genomes. By combining the *B. rapa* pan‐genome and population resequencing data, the pipeline generated the landscape of TIPs in *B. rapa* at a germplasm population scale.

### The landscapes of TE insertion polymorphisms in *B. rapa* genomes

**Figure 3:**
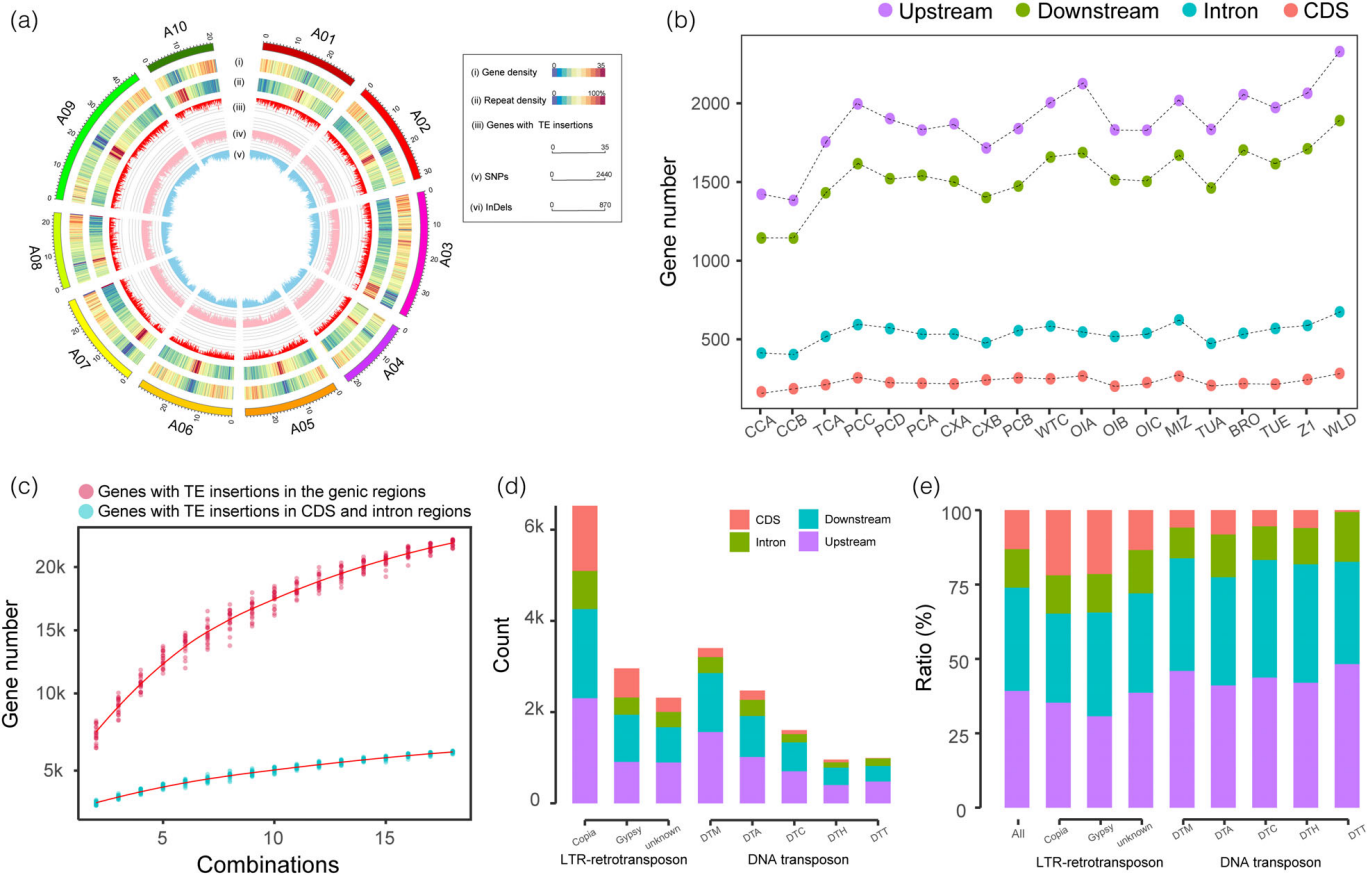
The distributions of transposable element (TE) insertions in the *B. rapa* genome. (a) Chromosomal distributions of genes (i) and TEs (ii) as well as TIP‐containing genes (iii) across the 10 chromosomes of the *B. rapa* Chiifu genome. Number of SNPs (iv) and InDels (v) in a sliding window of 100 kb. (b) The number of genes with TE insertions in different *B. rapa* genomes. (c) The number of genes with TE insertions in the genic regions after combinations of individuals. The different combinations were randomly selected from the 20 *B. rapa* genomes. (d, e) The numbers (d) and the ratios (e) of different TE insertions in the genic regions. Genic regions include 2 kb upstream and downstream of the gene body.

Based on the Chiifu genomic sequences, we divided the *B. rapa* pan‐genome into “aligned regions” and “unaligned regions” (Appendix S1). In total, we obtained 93 686 TE insertions in the “aligned regions,” and 96 657 TE insertions in the “unaligned regions,” occurred within 2 kb upstream and downstream of the gene body (this type of gene was called TIP‐containing gene). Specifically, we found 10 785 TIP‐containing genes (accounting for 23% of Chiifu genes) by taking the Chiifu sequence as the reference. These genes were widely distributed on the 10 chromosomes of *B. rapa* (Figure 3a). Furthermore, the 10 785 TIP‐containing genes corresponded to 2929–4819 genes with TE insertions in each of the other 19 genomes. Among these genes, 1382–2328 had TE insertions within the 2 kb upstream flanking region; 1142–1889 had TE insertions within the 2 kb downstream flanking region; 402–673 and 165–282 had TE insertions in intron and coding regions, respectively (Figure 3b). Modeling of the number of genes with TE insertions in the *B. rapa* pan‐genome showed that around 22 000 genes harbored TE insertions in the genic regions, and around 5400 genes harbored TE insertions in CDS and intron regions during *B. rapa* morphotype domestication (Figure 3c). The finding indicated extensive genes influenced by TE insertions in *B. rapa*.

Among different types of TIPs, LTR/Copia TIPs are more biased toward coding regions. Additionally, we annotated each TIP and extracted the two main TE families (LTR retrotransposons and DNA transposons) (Table S4). In total, there were 11 799 LTR‐retrotransposon TIPs and 9 434 DNA transposon TIPs in the Chiifu reference genome. The number of the LTR‐retrotransposon TIPs was 1.25 times that of the DNA transposon, which was significantly different from the ratio of LTR‐retrotransposons to DNA transposon content in the genome composition. The ratio of LTR‐retrotransposon to DNA transposon content was approximately 2.64 in different *B. rapa* genomes (Table S4). We found that the number of Copia TIPs was approximately 2.2 times the number of Gypsy TIPs (Table S4). However, the length of LTR/Gypsy repeat elements was almost twice that of LTR/Copia repeat elements (Table S1). Moreover, we calculated the numbers of TIPs that occurred 2 kb upstream of the gene, 2 kb downstream of the gene, introns, and coding regions (Figure 3d). We found that 21.8% of Copia TIPs and 21.46% of Gypsy TIPs inserted in the coding regions. These ratios were significantly higher than for the DNA transposon TIPs (the corresponding ratios of DTA, DTC, DTH, DTM, and DTT were 8.16%, 5.41%, 5.95%, 5.84%, and 0.60%, respectively) (Figure 3e), indicating that LTR repeat elements may have played a more crucial role in genome diversification.

### TE insertion polymorphisms contributed to large transcriptomic changes

**Figure 4:**
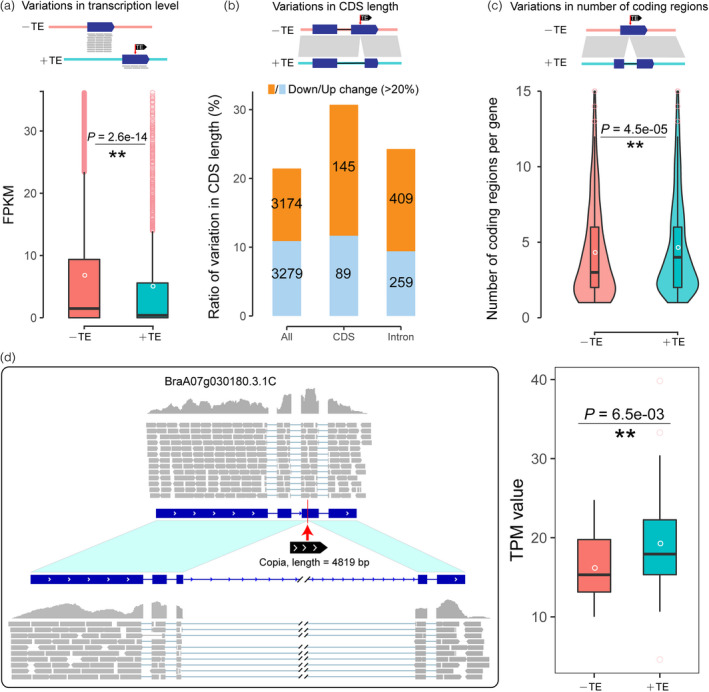
Transcriptional impacts of transposable element (TE) insertions. (a) Comparisons of genes with and without TE insertions in the *B. rapa* Chiifu genome. The ratio (number) of genes with significant changes in CDS length. Significant changes (down/up change >20%) in CDS length were calculated in all syntenic genes as well as genes with TIPs in coding and intron regions. Variations in CDS length (b) and number of coding regions per gene (c) between syntenic genes with and without TE insertions. We calculated syntenic gene pairs between a Chinese cabbage genome line (CCB) and a turnip rape line (OIB) and identified TIPs between syntenic gene pairs. (d) An example of a TE insertion acting as a new intron and being related to the changes in gene expression. The boxplot shows comparisons of expression levels of the BraA07g030180.3.1C with and without the LTR/Copia element insertion.

On average, genes with TE insertions had significantly lower transcription levels. For the reference Chiifu genome, 10 785 genes contained TIPs in genic regions. We found that the average transcriptional abundance of genes that harbored TE insertions was significantly lower than those of genes without TE insertions (*P* = 2.6e–14; Figure 4a), indicating that TIPs affected transcription.

To further explore the impacts of TIPs on transcriptomic changes, we took two different *B. rapa* genomes, CCB (Chinese cabbage) and OIB (rapid cycling), as an example to show the consequences of TIPs that appear in coding and intron regions of syntenic gene pairs. First, we identified 30 091 syntenic gene pairs between CCB and OIB genomes by taking orthologs in *A. thaliana* genome as a bridge. Among these gene pairs, the CDS lengths of 3174 and 3279 genes were significantly shorter (downward change >20%) and longer (upward change >20%). Furthermore, we investigated changes in CDS lengths of TIP‐containing genes. In total, there were 762 syntenic gene pairs with TIPs in their coding regions (TCR). Among them, the CDS lengths of 145 genes were significantly shorter, and 89 genes were significantly longer. Compared with changes in CDS length of all syntenic gene pairs, the coding sequences of TIP‐containing genes with TCR was significantly shorter (Fisher's exact test; *P* = 1.4e‐4) (Figure 4b). Meanwhile, we identified 2752 syntenic gene pairs with TIPs in their introns (TI), of which the CDS lengths of 409 genes were significantly shorter and 259 genes were significantly longer (Figure 4b), the coding sequences of TIP‐containing genes with TI was also significantly shorter (Fisher's exact test; *P* = 3.3e‐09). The same pattern was also observed in other accession combinations (Table S5).

We further analyzed the relationship between TIPs and varied number of coding regions per gene. We found that the average coding regions per gene was significantly higher than that of genes without TIPs (Figure 4c, *P* = 4.5e‐05), implying that TE insertions may contribute to modifying gene structure by being incorporated as new introns. Furthermore, by manually analyzing microsynteny and expression patterns of syntenic gene pairs with TIPs, we provided an example that the TE insertions acted as new introns and thereby may affect transcription. An LTR/Copia repeat element in length of 4819 bp inserted in exon 3 of the BraA07g030180.3.1C gene (Figure 4d). The orthologous gene of *BraA07g030180.3.1C* in *A. thaliana* is *PATL1*, which is a carrier protein that may be involved in membrane‐trafficking events associated with cell plate formation during cytokinesis. We checked the expression of the BraA07g030180.3.1C gene in 86 *B. rapa* accessions consisting of 42 Chinese cabbages and 44 non‐Chinese cabbages. The transcriptome data were available from our previous study (Cheng *et al*., 2016). We found that the average transcript abundance of the gene with the LTR/Copia repeat element insertion was significantly higher than that of the gene without this LTR/Copia (Figure 4d). These results further highlight the large impacts of TIPs on transcriptomic changes.

### TE insertion polymorphisms are tightly associated with the domestication of *B. rapa* morphotypes

**Figure 5:**
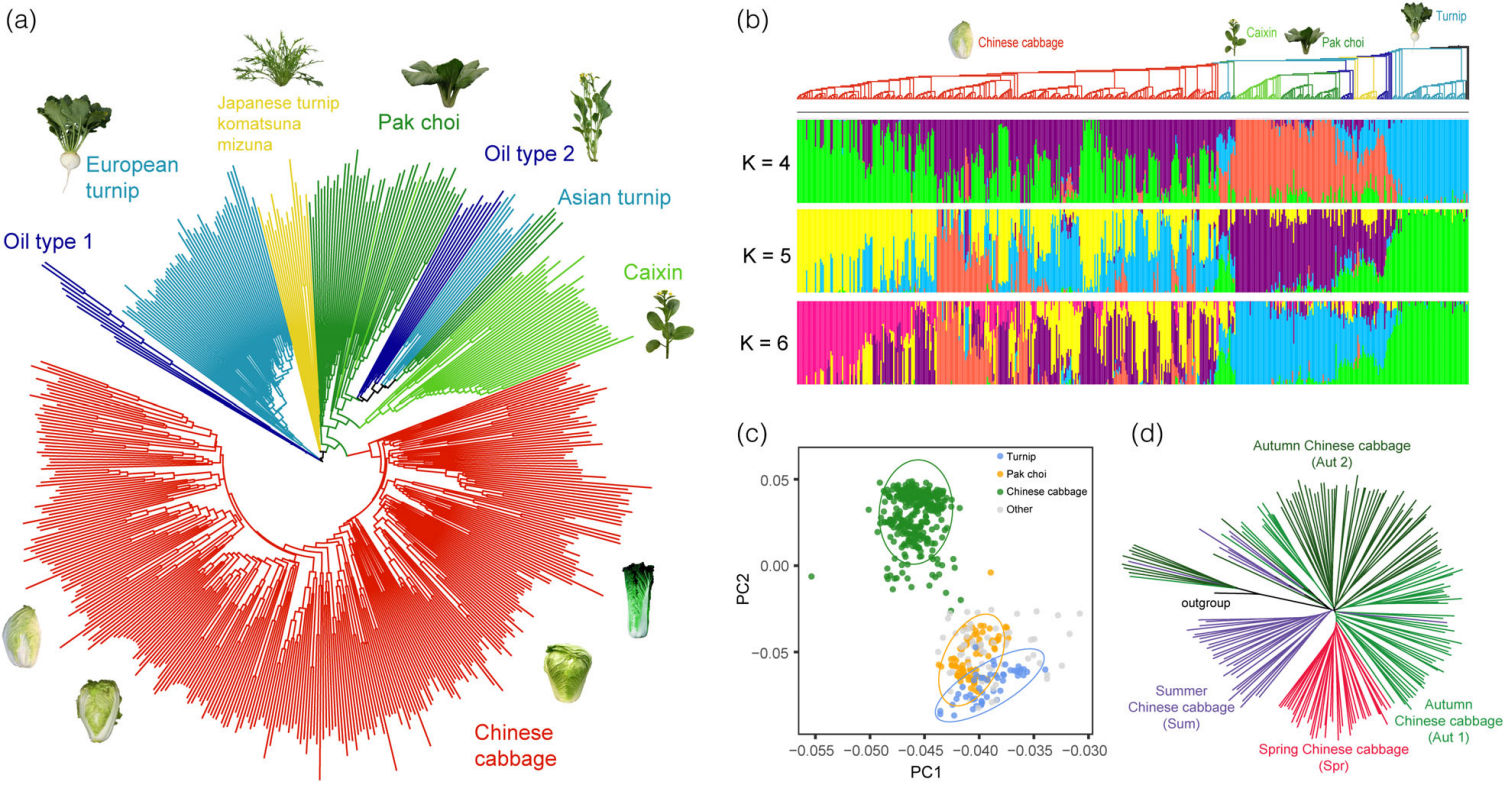
Transposable element insertion polymorphisms (TIPs) associated with *B. rapa* morphotype domestication and selective development. (a) Phylogenetic tree of 524 *B. rapa* accessions using TIPs. Pictures placed beside each clade show typical morphotypes for the corresponding groups. (b) The genetic components calculated for the 524 *B. rapa* accessions using TIPs. (c) PCA plot of 524 *B. rapa* accessions using TIPs. (d) Phylogenetic tree of 192 Chinese cabbages using TIPs. The turnip was employed as the outgroup. The three ecotypes of spring, summer, and autumn Chinese cabbage were empirically divided by Su *et al*. (2018).

To explore the impacts of TIPs on the domestication of morphotypes in *B. rapa*, we investigated TE insertions in 524 diverse *B. rapa* genomes, including accessions from morphotypes of Chinese cabbage, turnip, turnip rape, pak choi, and caixin (Cai *et al*., 2021). The phylogenetic tree constructed by TIPs in 524 *B. rapa* genomes clearly divided the *B. rapa* species into the groups of Chinese cabbage, turnip, pak choi, and others (Figure 5a), consistent with our previous phylogenetic tree constructed using whole‐genome SNPs (Cai *et al*., 2021). Meanwhile, the genetic components and PCA analysis using TIPs in 524 *B. rapa* genomes also revealed that TIPs were tightly associated with *B. rapa* morphotype domestication (Figure 5b,c).

Furthermore, we observed that TIPs were associated with the selective development of Chinese cabbage during modern breeding. We collected 192 Chinese cabbage accessions representing four sub‐ecotypes according to growing season (spring, summer, autumn 1, and autumn 2) (Su *et al*., 2018). The TIP‐phylogenetic tree (Figure 5d) clearly distinguished clusters of each seasonal ecotypes; accessions from the spring ecotype and summer ecotype were closely and separately clustered, indicating involvement of the TIPs in formation of different Chinese cabbage ecotypes.

### TE insertion polymorphisms have played a crucial role in the domestication of morphotypes in *B. rapa*

**Figure 6:**
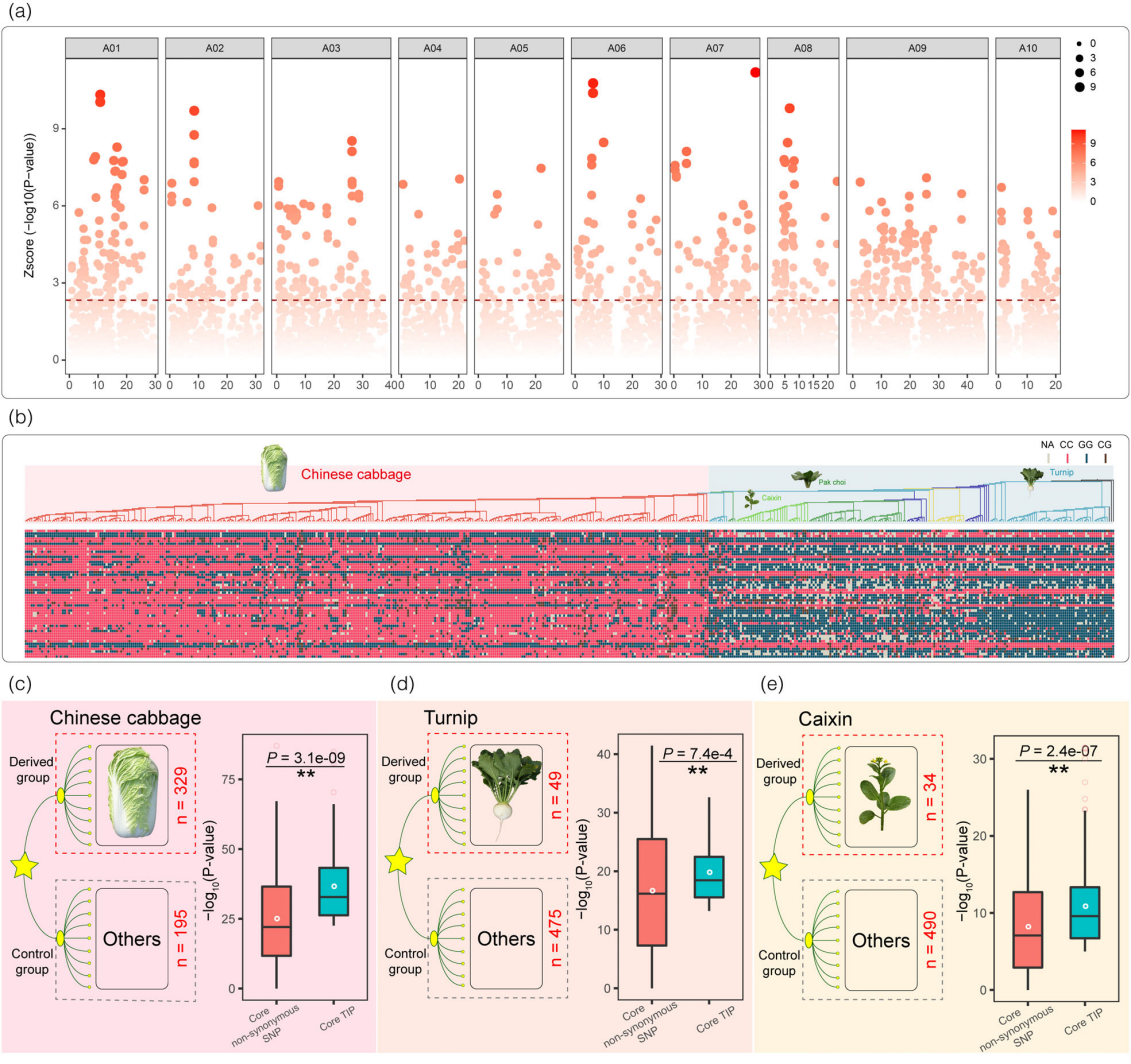
Transposable element (TE) insertion polymorphisms (TIPs) played a crucial role in the domestication of morphotypes in *B. rapa*. (a) Genomic signatures of selection in genomes of Chinese cabbage using whole‐genome TIPs. We calculated the frequency of each TE insertion in the heading and non‐heading *B. rapa* populations and conducted Fisher’s exact test for each TIP. After that, each P‐value was normalized by ‐log10 and z‐score. (b) The genotypes for the top 50 selection signatures in the heading and non‐heading populations in *B. rapa*. The phylogenetic tree of 524 *B. rapa* accessions was reported in our previous *B. rapa* pan‐genome analysis. CC indicates that the genotype in the corresponding accession was consistent with the reference genome, and GG indicates that the genotype in the accession was different from the reference genome, while missing loci (NN) and heterozygous loci (CG) are filled with gray and brown. (c–e) Comparison of selection pressure between TIPs and non‐synonymous SNPs in TIP‐containing candidate genes during the domestication of Chinese cabbage (c), turnip (d), and caixin (e) morphotypes. Accessions from the target morphotypes (Chinese cabbage, turnip, and caixin) are respectively set as derived groups, and all accessions except the target morphotypes were set as control groups. The red number on the right side of each picture shows the number of accessions of the target morphotype.

To identify those TIPs selected during domestication, we inspected the genomic distributions of TIPs in 524 diverse *B. rapa* accessions. Trait domestication analysis was carried out on three representative *B. rapa* morphotypes, Chinese cabbage (leafy head), pak choi (nonheading), and turnip (swollen hypocotyls with varying degrees of root and stem). Accessions with the target morphotype formed the derived group, and all other accessions were taken as the control group. Enrichment of each TIP in the “aligned regions” of the two groups was evaluated using Fisher's exact test. This identified 812 TIPs corresponding to 682 candidate genes that were related to the domestication of Chinese cabbage (Figure 6a,b; Table S6). Furthermore, we found 772 TIPs corresponding to 755 genes related to the domestication of pak choi and 880 TIPs with 742 genes related to the domestication of turnip (Tables S7 and S8). Similarly, we found that TIPs in the “unaligned regions” were also tightly associated with the domestication of the three morphotypes (Figure S3). In total, 394, 408, and 464 TIPs corresponding to 411, 437, and 489 genes, respectively (Tables S9–S11), were related to the domestication of Chinese cabbage, pak choi, and turnip.

To further evaluate the contributions of TIPs in the domestication of different *B. rapa* morphotypes, we calculated the enrichment of TIPs and non‐synonymous SNPs that occurred in the candidate gene regions. During the domestication of the *B. rapa* heading group, 128 out of 682 candidate genes harbored TIPs in their gene regions (introns and coding regions) in the aligned regions (Table S6). We extracted TIPs and nonsynonymous SNPs in the gene regions and calculated the enrichment of each TIP or nonsynonymous SNP in the heading and nonheading groups using Fisher's exact test. The TIP or nonsynonymous SNP with the smallest *P*‐value was defined as the core TIP or the core nonsynonymous SNP, respectively. We found that nine out of 128 TIP‐containing candidate genes did not harbor SNPs, and the average *P*‐value of core TIPs was significantly lower than that of core nonsynonymous SNPs (Figure 6c, Table S12, Paired *t*‐test, *P* = 3.1e–09). This result suggested these TIPs having been selected by a stronger pressure than nonsynonymous SNPs. Meanwhile, the results also indicated that these TIPs contributed greatly in the same level or even higher level compared to that of nonsynonymous SNPs in the domestication of the heading morphotype. Additionally, the average *P*‐value of core TIPs was also significantly lower than that of core nonsynonymous SNPs in the domestication of the turnip and caixin morphotypes (Figure 6d,e, Tables S13 and S14, Paired *t*‐test, *P*_turnip_ = 7.4e‐04, *P*_caixin_ = 2.4e‐07), also consistent with the above results.

### TE insertion polymorphism‐containing genes strongly selected during diverse crop domestication in *B. rapa*

Among the above candidates, we provided examples that four TIP‐containing genes might be involved in climate change adaptation, promoting the formation of diverse crops in *B. rapa*.

**Figure 7:**
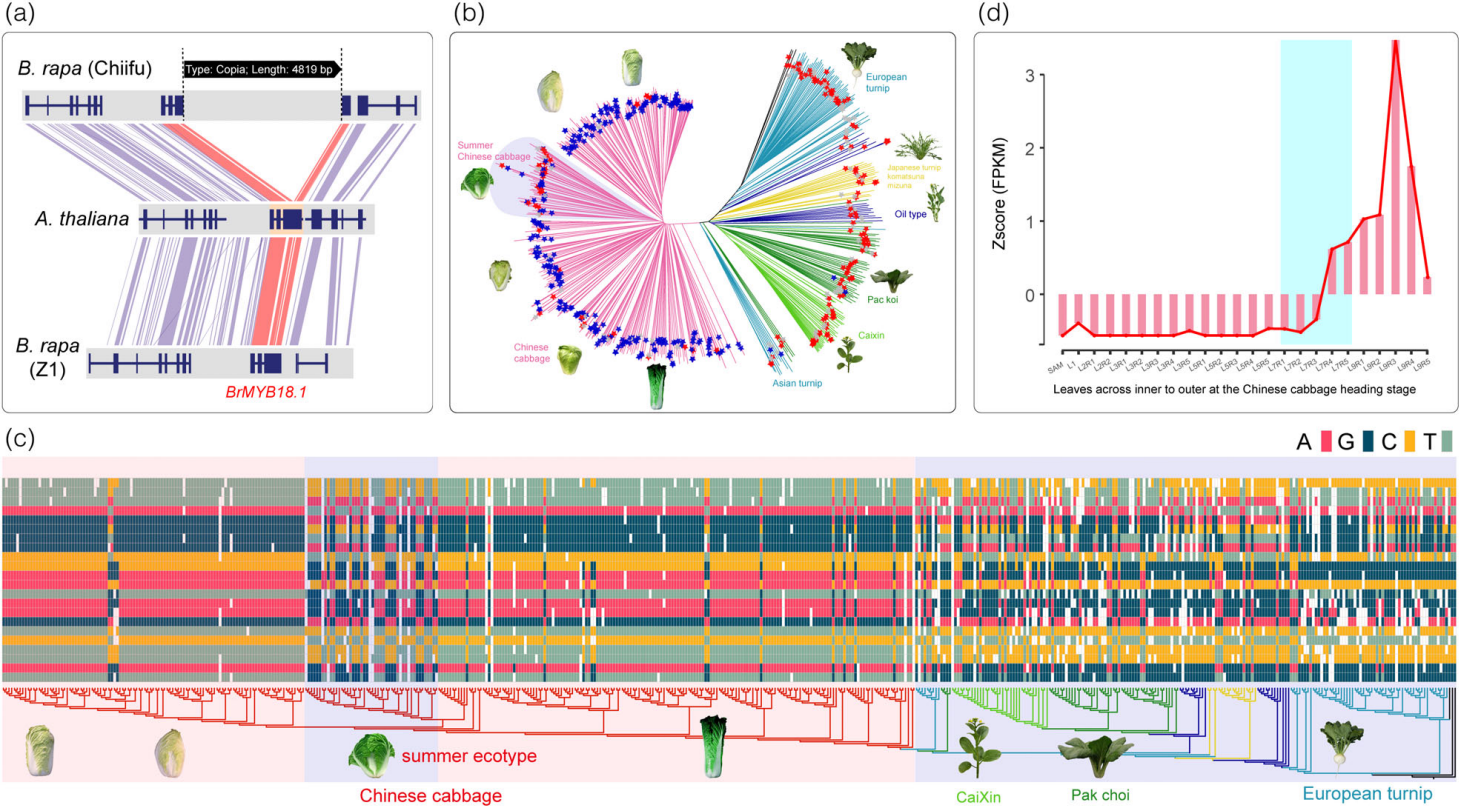
The TIP‐containing gene *BrMYB18.1* was under strong selection during the domestication of Chinese cabbage. (a) The Copia element inserted in coding regions of *BrMYB18.1* in the Chinese cabbage genome. (b) The distribution of the insertion of the Copia element in 524 *B. rapa* genomes. The tree was constructed using whole‐genome SNPs, reported in our previous pan‐genome analysis (Cai *et al*., 2021). The blue and red stars indicate the corresponding accessions with and without the transposable element (TE) insertion, respectively. (c) The distribution of haplotypes in the *BrMYB18.1* gene region in 524 genomes. Homozygous sites of AA, CC, GG, and TT are filled using different colors as described in the figure, while missing loci (NN) and heterozygous loci (Hetero) are not filled with color. (d) The expression patterns of the *BrMYB18.1* gene in the Chinese cabbage leaves. L1–L9 represented different leaves from inner to outer at the Chinese cabbage heading stage. R1–R5 represented different regions of each leaf as described by Guo *et al*. (2021).

*Arabidopsis MYB18* gene encodes a nuclear protein with strong homology with the R2R3–MYB family (Ballesteros *et al*., 2001), and is a positive regulator of the phyA photoresponse (Jang *et al*., 2013). An orthologue has also been reported as regulating leaf rolling in rice (Zhang *et al*., 2016). We detected a 4819 bp LTR/Copia element inserted in the third exon of *BrMYB18.1*, an orthologue of the *MYB18* gene (Figure 7a). Furthermore, we calculated the distribution frequency of LTR/Copia inserted *MYB18.1* in the 524 *B. rapa* genomes. The result turned out to be a significant enrichment of the LTR/Copia‐inserted *BrMYB18.1* in the heading population (Fisher's exact test; *P* = 8.3e‐67). Of the 329 heading accessions, 265 (80%) harbored the LTR/Copia insertion while, among the 195 nonheading accessions, only four (2%) harbored the LTR/Copia insertion (Figure 7b). Meanwhile, we extracted SNPs in the genic region of *BrMYB18.1*, and concatenated these to form the *BrMYB18.1* gene haplotype. We found that the heading group displayed a uniform *BrMYB18.1* (Figure 7c), also supporting the suggestion that the *BrMYB18.1* was under strong selection during the domestication of the heading group. Moreover, we analyzed the expression levels of the TIP‐containing *BrMYB18.1* gene in the leaves from inner to outer at Chinese cabbage heading stage; the expression of this gene suddenly enhanced significantly in the leaf L7 and keeping high level expression in the afterward leaves (Figure 7d). This specific leaf was considered to be the key transition leaf during the leafy head formation (Guo *et al*., 2021). Unexpectedly, distribution of either the TIP or the gene haplotype of *BrMYB18.1* in summer Chinese cabbage was like the nonheading morphotypes, suggesting that this locus had been re‐introduced into summer Chinese cabbage during the modern breeding.

We identified a strongly selected TIP that altered *BrFLOR1.2* gene structure. In *A. thaliana FLOR1.2* interacts with a MADS box transcription factor to delay flowering (Torti *et al*., 2012). In *B. rapa* genomes, a 4716 bp LTR/Copia element inserted in the first exon of *BrFLOR1.2* divided the first exon into two exons, acting as a new intron itself (Figure S4). While calculating the distribution of the 4716 bp LTR/Copia insertion among the 524 genomes, we found that the *BrFLOR1.2* insertion was significantly enriched in the caixin morphotype (Figure S4; Fisher's exact test; *P* = 1.5e‐31). In addition, we constructed the *BrFLOR1.2* haplotype and found that the caixin morphotype displayed a uniform haplotype (Figure S5), supporting *BrFLOR1.2* being strongly selected during the domestication of the caixin morphotype. Caixin bolts rapidly without the requirement for vernalization. We deduced that the Copia insertion in *BrFLOR1.2* might be related to early flowering and that this locus was selected during the breeding of the vegetable crop caixin.

Flowering time is the most import trait associated with domestication and artificial selection of genes implicated in morphological diversification of crops. Having or not the requirement of vernalization, or the cold extent and duration extremely varied among different *B. rapa* subspecies or ecotypes. *VRN1* plays an important role in repressing the expression of *FLOWERING LOCUS C* (*FLC*) during vernalization in *A. thaliana* (Levy *et al*., 2002). *AtVRN1* encodes a protein with a DNA‐binding domain and PEST region involved in the stable repression of *FLC* (Levy *et al*., 2002). The expression level of *BrVRN1.2* was significantly increased upon vernalization in *B. rapa* (Figure S6). By analyzing the sequence of *BrVRN1.2*, we identified a 5488 bp LTR/Copia element insertion in the upstream region of *BrVRN1.2* in CXA and CXB genomes (both were from caixin). Furthermore, we found that the insertion was significantly enriched in the groups of caixin and summer Chinese cabbage (Figure S6; Fisher's exact test; *P*_(caixin)_ = 6.5e‐25; *P*_(summer Chinese cabbage)_ = 2.0e‐08). Additionally, gene haplotype analysis also supported the hypothesis that the TIP‐containing *BrVRN1.2* was under strong selection during the domestication of the caixin and summer Chinese cabbage morphotypes (Figure S7). Importantly, it is worth noting that initiation of flowering in the two vegetable crops caixin and summer Chinese cabbage does not require vernalization.

Additionally, we identified a 3023 bp LTR element inserted in the second intron of *BrFT2* in a yellow sarson line (OIB), which was absent in a caixin line (CXB) (Figure S8). Both accessions do not require vernalization. Tissue‐specific transcriptome sequencing showed that *BrFT2* transcript abundance in L58 was 44.5 times that of R‐o‐18 in leaves, 29.2x in roots, and 18.2x in flowers. The FPKM values of the *BrFT2* gene with the LTR insertion in the leaves, roots, and flowers of the R‐o‐18 line were 4.67, 3.42, and 0.55, respectively, indicating that TE insertion resulted in minimal *BrFT2* expression (Figure S8). Previously, we concluded that a TE insertion of unknown length resulted in failed transcription of the *BrFT2* gene, contributing to delayed flowering (Zhang *et al*., 2015). Here, we determined that the TE insertion length was 3023 bp, and provided transcript data to further support TIP as an important source of domesticated phenotypic variation.

## Discussion

### Transposable elements insertion polymorphism has been a hidden source of phenotypic variants in the past few decades

Overall, our results have shed new light on the important role of TE insertions in phenotypic variation and crop morphotype domestication in *B. rapa*. Two‐thirds of TE insertions studied in the Arabidopsis gene pool have previously been found not to be in linkage disequilibrium with nearby SNPs (Stuart *et al*., 2016), indicating that TE insertion was a potential source of novel genetic diversity. Additionally, TE insertion is recognized as an important source of phenotypic variants. In tomato genomes, it was reported that TE insertions were linked to fruit color and leaf morphology (Busch *et al*., 2011; Dominguez *et al*., 2020; Fray and Grierson, 1993). In *Brassica napus* genomes, an LTR‐insertion is linked with resistance to pod shattering (Liu *et al*., 2020a), and hAT, MITE, and LINE insertions are associated with flowering time variations (Song *et al*., 2020).

A large body of work has shown that TE insertions are an important diversifying force, resulting in speciation, adaptation, and the domestication of crucial traits (Baduel *et al*., 2019; Carpentier *et al*., 2019; Liu *et al*., 2020a; Quadrana *et al*., 2016; Serrato‐Capuchina and Matute, 2018; Song *et al*., 2020). These radical outcomes can be explained by different mechanisms, including TE bursts in intergenic regions that have led to rapid changes in genome size and genome structure (Kim *et al*., 2017; Vitte and Panaud, 2005), as well as biased TE insertions occurring near or within genes (Liu *et al*., 2020a; Song *et al*., 2020; Zhang *et al*., 2015). TE insertions that occur in the coding region can directly alter the gene structure, those that occur within introns and flanking regions can regulate gene expression (Chuong *et al*., 2017; Sultana *et al*., 2017). It should be noted that TE insertions are different from other SVs, because TEs carry multiple *cis*‐regulatory sequences, and can therefore modify gene network and epigenetic regulation (Stuart *et al*., 2016; Sultana *et al*., 2017; Uzunovic *et al*., 2019). In this study, we found an interesting phenomenon, that is, that TE insertions act as new introns and may also cause significant changes in gene expression (Figure 4d). Meanwhile, we demonstrated that the TIPs in the TIP‐containing candidate genes experienced a stronger selection pressure than the nonsynonymous SNPs during *B. rapa* morphotype domestication. We deduced that TIPs have played an important role in *B. rapa* morphotype domestication, since non‐synonymous SNPs are usually considered to be involved in changes of gene function and TIPs in TIP‐containing candidate genes are under stronger selection pressure than nonsynonymous SNPs. It was also found that TIPs exhibited larger phenotypic effects in tomato than SNPs (Dominguez *et al*., 2020). Our results reinvigorated the idea that TE insertions were pervasively co‐opted for the modification of host genes.

### The combination of pan‐genome analysis and population‐scale resequencing enhances understanding of complex genomic variations

Systematically exploring the impacts of TE insertions on trait domestication is poorly documented since it is challenging to fully identify TE insertions using short reads. Previously, the pipelines of SPLITREADER (Baduel *et al*., 2021), MELT (Gardner *et al*., 2017), RelocaTE2 (Chen *et al*., 2017), and TEPID (Stuart *et al*., 2016) were developed to detect TE insertions by mapping the short reads onto the reference genome. Obviously, these algorithms cannot deal with complex TE insertions due to the limitations of short reads and identification of TE insertions present in the nonreference sequences. Here, we proposed and demonstrated a new method employing the combination of pan‐genome analysis and population‐scale resequencing. The advantage of this method is that the usage of pan‐genome guarantees accurate determination of TE insertions, including TE insertions in the reference and nonreference regions. Thereafter, the short reads from a large‐scale re‐sequencing could be used to genotype each TE insertion. Since all TE insertions have been verified by the assemblies that were constructed by long reads, it is feasible to genotype TE insertions using short reads. The combination of pan‐genome analysis and population resequencing enhances the understanding of TIPs. Meanwhile, the pipeline also has limitations, for example, it ignores TIPs in intergenic regions.

A large amount of resequencing data for crops such as rice, soybeans, tomatoes, and *B. rapa* has been accumulated. Hundreds of germplasm accessions have been resequenced (Cai *et al*., 2021; Gao *et al*., 2019; Liu *et al*., 2020b; Wang *et al*., 2018). In contrast, only a few dozen representative varieties for a species have been *de novo* assembled, because population‐scale *de novo* assembly is still expensive and consumes a large amount of computer resources. Therefore, it is essential to make full use of the advantages of pan‐genome analysis and population‐scale resequencing to explore complex SVs. The pipeline we developed in this study efficiently use a pan‐genome data framework to identify complex genomic variations, and then employ population‐scale resequencing reads to genotype each genomic variation.

In this work, we developed an effective process for the systematic identification of TIPs. This process will promote in‐depth mining of pan‐genome and population resequencing data. Additionally, the large number of TIPs we identified here are relevant to *B. rapa* morphotype diversity, revealing their important role in trait domestication with awareness of the implications for breeding. Collectively, our work has reinvigorated the importance of TIPs in the domestication of crucial agronomic traits of *B. rapa*.

## Methods

### Identification of transposable elements in *B. rapa* genomes

A total of 20 *B. rapa* assemblies were collected from the *B. rapa* pan‐genome analysis (Cai *et al*., 2021) and two recent studies on the pak choi genomes (Li *et al*., 2020, 2021). The 20 *B. rapa* genomes were used to construct the *B. rapa* TE library and identify TE insertions. First, the EDTA pipeline (v1.9.7) (Pritchard *et al*., 2000) was used to predict TEs and construct a nonredundant TE library for the *B. rapa* species using default parameters. The DNA transposons and LTR retrotransposons were extracted from the output results of the EDTA pipeline, including the Copia‐like, Gypsy‐like LTR‐RTs, and six other DNA transposons (Helitron, DTM, DTA, DTC, DTH, and DTT) (Feschotte and Pritham, 2007). After that, we used the RepeatMasker (version: open‐4.0.7) (Tarailo‐Graovac and Chen, 2009) software to identify the total TE content in each of the 20 genomes using our constructed TE library.

### Identification of TE insertion polymorphisms in *B. rapa*

Identification of TE insertion polymorphism between different *B. rapa* genomes. First, we used the Chiifu genome as the reference, and broke the remaining 19 genome sequences into contigs. Genome contigs were aligned to the Chiifu genome for identifying insertions and deletions between the reference and each of the 19 genomes using the smartie‐sv pipeline (Kronenberg *et al*., 2018). Next, we removed insertions and deletions that contained gaps. Based on the same method, each of the 20 *B. rapa* genomes was used as the reference to comprehensively identify insertions and deletions in the *B. rapa* pan‐genome. After that, sequences of all insertions and deletions were mapped to the *B. rapa* TE library using blastn based on the “80–80 rule.” If both the identity and coverage of each deletion (or insertion) reached 80%, then the deletion was defined as a TE insertion. Using this strategy, we determined all TIPs in the *B. rapa* pan‐genome. Furthermore, we extracted TIPs that appeared in the genic regions (including 2 kb upstream and downstream of the gene body). Additionally, “aligned regions” and “unaligned regions” we proposed in this study were also calculated by the smartie‐sv pipeline (Kronenberg *et al*., 2018) with default parameters.

### Genotyping of TE insertion polymorphisms using population‐scale short reads

First, we extracted 1 kb upstream and downstream sequences of each TE insertion. The TE insertion sequence together with their flanking sequences was further used for genotyping TE insertions. Next, all short reads of 524 *B. rapa* accessions were mapped onto the TE insertion and their flanking sequences using the “bwa mem” algorithm (version: 0.7.17‐r1188) with the parameters “‐T 20 –Y” (Li and Durbin, 2009). A read being able to be aligned to the TE insertion and flanking sequences and the alignment length being greater than 20 bp were used for genotyping. After that, we checked whether the left and the right TE insertion sites were covered by the short reads. Since each TE insertion had been confirmed using the assembled genomes, if one or two TE insertion boundaries were covered by short reads, we believed that the accession also harbored the same TE insertion. Based on this principle, four types of mapping patterns (described as “CC” in Figure 2) supported the accession as harboring the TE insertion. If one or two insertion boundaries were covered by short reads, the accession harbored the TE insertion. In paired‐end reads, if one was aligned to the flanking sequences (1 kb upstream/downstream) and the other was aligned to the TE sequence, then the accession also harbored the same TE insertion. Additionally, if the paired‐end reads all mapped onto the flanking sequences, this indicated that the accession did not harbor the TE insertion.

### Validation of transposable element insertions

We used two methods to verify the reliability of TE insertions. First, we aligned the PacBio reads to the reference genome using minimap2 (version: r1061) (Li, 2018) with the parameters “‐ax map‐pb.” Then, we used IGV (Robinson *et al*., 2011) to present the alignment results and checked the TE insertions by hand. Second, we also used short reads to verify TE insertions. The short reads were mapped onto the reference sequences using minimap2 (version: r1061) (Li, 2018) with the parameters “‐ax sr.” Then, we extracted the mapping results of paired‐end reads and calculated the distribution of paired‐end reads on the target region. If the insertion size of the paired‐end reads in the target region was unusual, and the insertion size was equal to the sum of the length of TE insertion and the average insertion size of the total paired‐end reads, then we concluded that the accession harbored the same TE insertion (Figure S9).

### Phylogenetic analysis

To construct the phylogenetic tree of the 20 *B. rapa* accessions, we selected the *B. oleracea* (JZS) genome (Cai *et al*., 2021) as the outgroup. First, we used OrthoFinder (version: 2.5.2) (Emms and Kelly, 2019) to detect single‐copy genes between *B. oleracea* and the 20 *B. rapa* genomes. In total, we detected 6798 single‐copy genes, and the phylogenetic tree was calculated based on these single‐copy genes. Second, we extracted the coding sequences of these single‐copy genes and used MAFFT (version v7.402) (Katoh *et al*., 2005) to conduct multiple sequences alignments. Gblock (version: v0.91b) (Talavera and Castresana, 2007) with the parameters “‐t = p ‐b4 = 5 ‐b5 = h” was further used to extract the conserved sequences among the 20 *B. rapa* genomes and one *B. oleracea* genome. Finally, the phylogenetic tree was constructed using RAxML (version v8.2.12) (Stamatakis, 2014) with 100 bootstrap replicates.

### TE insertion polymorphism‐based population analysis

After genotyping TE insertions in each of the 524 *B. rapa* genomes, we merged TE insertions in 524 genomes and investigated the relationship between TIPs and the domestication of morphotypes. First, we used “CC” and “GG” to represent TE insertions in each accession. “CC” meant that there was a TE insertion, and “GG” meant that there was no TE insertion, while missing loci were encoded as NN. Second, we removed homozygous loci with MAF ≤ 0.05. Then, phylogenetic analysis, structure analysis, and PCA analysis were conducted based on this dataset. We used Perl script to calculate a distance matrix for each pair of TIPs, and then used PHYLIP (version: v3.69) (Retief, 2000) to construct a neighbor‐joining phylogenetic tree of the 425 *B. rapa* accessions. Population structure analysis was conducted using STRUCTURE (version: 2.3.4) (Pritchard *et al*., 2000) with K = 2–10 clusters, and we selected the results with K = 4 and K = 5 to represent the genetic structures of the 524 *B. rapa* accessions. PCA analysis of TIPs in the 524 *B. rapa* genomes was conducted using plink (version: v1.90b4) (http://www.cog‐genomics.org/plink/1.9/) (Chang *et al*., 2015) with the parameters “—noweb ‐‐pca 20.”

## Competing interests

Authors declare that they have no competing interests.

## Author contributions

JW and XWW conceived the research and designed the experiments. XC performed the experiments. XC analyzed the data. XC, JW, and XWW wrote the manuscript. GJK, RML, and JLL improved the manuscript. JW and XWW contributed equally. All authors of this paper read and approved the final manuscript.

## Code availability

Code used for identifying TIPs in the *B. rapa* pan‐genome and genotyping TIPs in 524 *B. rapa* genomes are available at GitHub (https://github.com/caixu0518/ITIPs).

## Supporting information


**Figure S1** Images of the different representative morphotypes of *B. rapa*.
**Figure S2** Correlation analysis between transposable element (TE) content and the assembled genome size of each *B. rapa* accession.
**Figure S3** The genotypes for the top 50 selection signatures in the heading and nonheading populations in *B. rapa* using transposable element insertion polymorphisms (TIPs) in the unaligned regions.
**Figure S4** A Copia insertion in *BrFLOR1.2* was associated with the domestication of caixin.
**Figure S5** The distribution of haplotypes in the *BrFLOR1.2* gene region in 524 genomes.
**Figure S6** A Copia insertion in *BrVRN1.2* was associated with the domestication of caixin and summer Chinese cabbage.
**Figure S7** The distribution of haplotypes in the *BrVRN1.2* gene region in 524 genomes.
**Figure S8** An LTR insertion in *BrFT2* gene.
**Figure S9** The validation of transposable element (TE) insertions among different *B. rapa* genomes based on the insertion sizes of paired‐end reads.

**Table S1** Statistics of transposable elements (TEs) in each of the 20 *B. rapa* genomes.
**Table S2** The number of insertions and deletions using each of the 20 *B. rapa* genomes as the reference.
**Table S3** Statistics of aligned and unaligned regions using Chiifu as the reference.
**Table S4** The numbers and lengths of different types of transposable element insertion polymorphisms (TIPs) in the *B. rapa* genome using different genomes as the references.
**Table S5** Variations in CDS length of coding regions per gene between syntenic genes with TIPs.
**Table S6** Selection signals in Chinese cabbage morphotype in the aligned regions.
**Table S7** Selection signals in the pak choi morphotype in the aligned regions.
**Table S8** Selection signals in the turnip morphotype in the aligned regions.
**Table S9** Selection signals in Chinese cabbage using transposable element insertion polymorphisms (TIPs) in unaligned regions.
**Table S10** Selection signals in pak choi using transposable element insertion polymorphisms (TIPs) in unaligned regions.
**Table S11** Selection signals in turnip using transposable element insertion polymorphisms (TIPs) in unaligned regions.
**Table S12** The core TIP and core nonsynonymous SNP in TIP‐containing candidate genes during the domestication of Chinese cabbage.
**Table S13** The core transposable element insertion polymorphism (TIP) and core nonsynonymous SNP in TIP‐containing candidate genes during the domestication of turnip.
**Table S14** The core transposable element insertion polymorphism (TIP) and core nonsynonymous SNP in TIP‐containing candidate genes during the domestication of caixin.

**Appendix S1** The pipeline to retrieve population‐scale TIPs.

## Data Availability

Transcriptome sequencing reads that were generated from roots, leaves, and flowers of a yellow sarson line (OIB) and a caixin line (CXB) have been deposited in NCBI under the accession number PRJNA758599. The TE insertions in the 524 *B. rapa* genomes have been submitted to Figshare (https://figshare.com/articles/dataset/BraTIPs/16691485).
