# Supplementary material for: Transposable element insertion: a hidden major source of domesticated phenotypic variation in Brassica rapa
Source: Plant Biotechnol J. 2022 Mar 18;20(7):1298–310. doi: 10.1111/pbi.13807 (PMC9241368; doi:10.1111/pbi.13807)
Supplement: Supplementary file 1 — Figure S1 Images of the different representative morphotypes of B. rapa. Figure S2 Correlation analysis between transposable element (TE) content and the assembled genome size of each B. rapa accession. Figure S3 The genotypes for the top 50 selection signatures in the heading and nonheading populations in B. rapa using transposable element insertion polymorphisms (TIPs) in the unaligned regions. Figure S4 A Copia insertion in BrFLOR1.2 was associated with the domestication of caixin. Figure S5 The distribution of haplotypes in the BrFLOR1.2 gene region in 524 genomes. Figure S6 A Copia insertion in BrVRN1.2 was associated with the domestication of caixin and summer Chinese cabbage. Figure S7 The distribution of haplotypes in the BrVRN1.2 gene region in 524 genomes. Figure S8 An LTR insertion in BrFT2 gene. Figure S9 The validation of transposable element (TE) insertions among different B. rapa genomes based on the insertion sizes of paired‐end reads. [file PBI-20-1298-s001.doc]

**Supplementary figures**


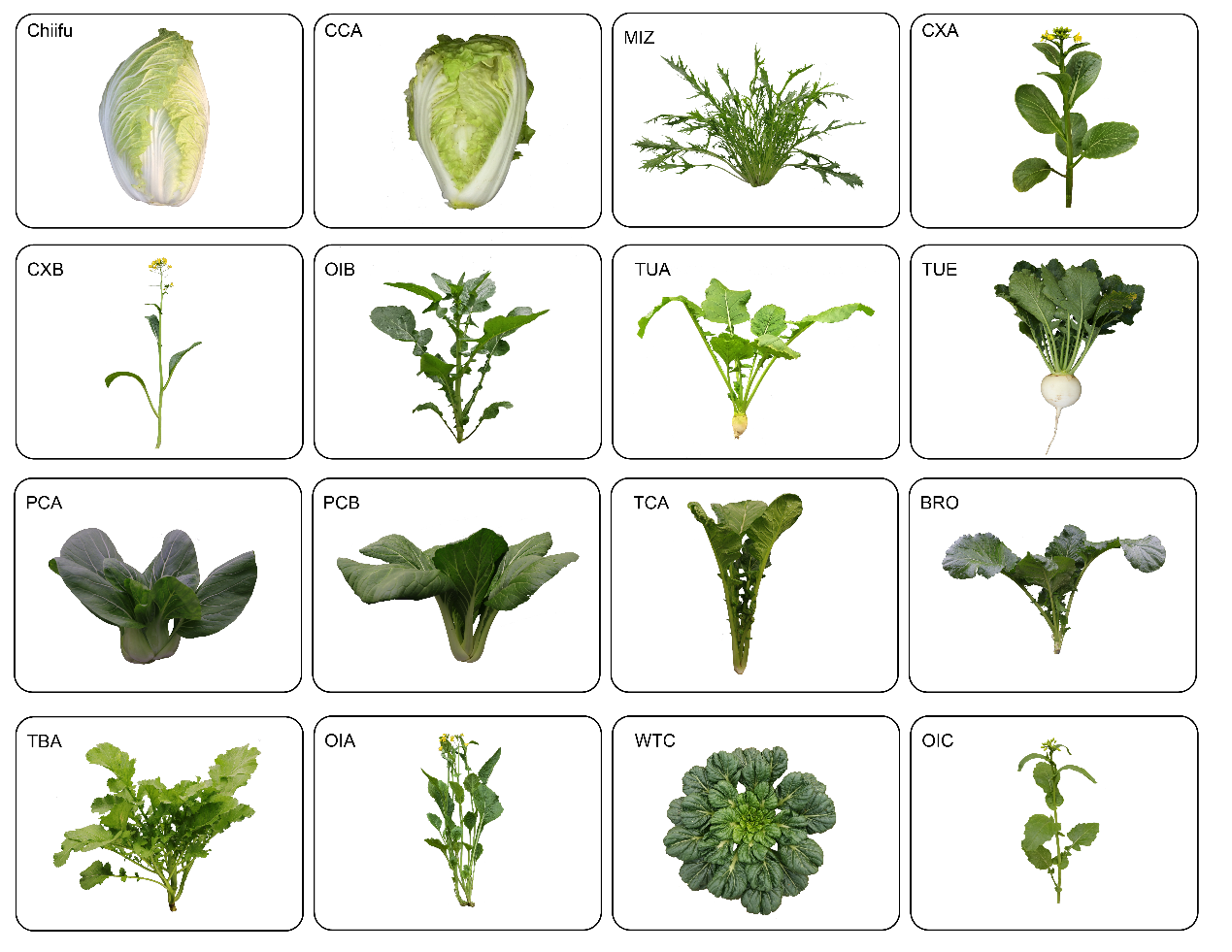


**Fig. S1 Images of the different representative morphotypes of *B. rapa*.**

**
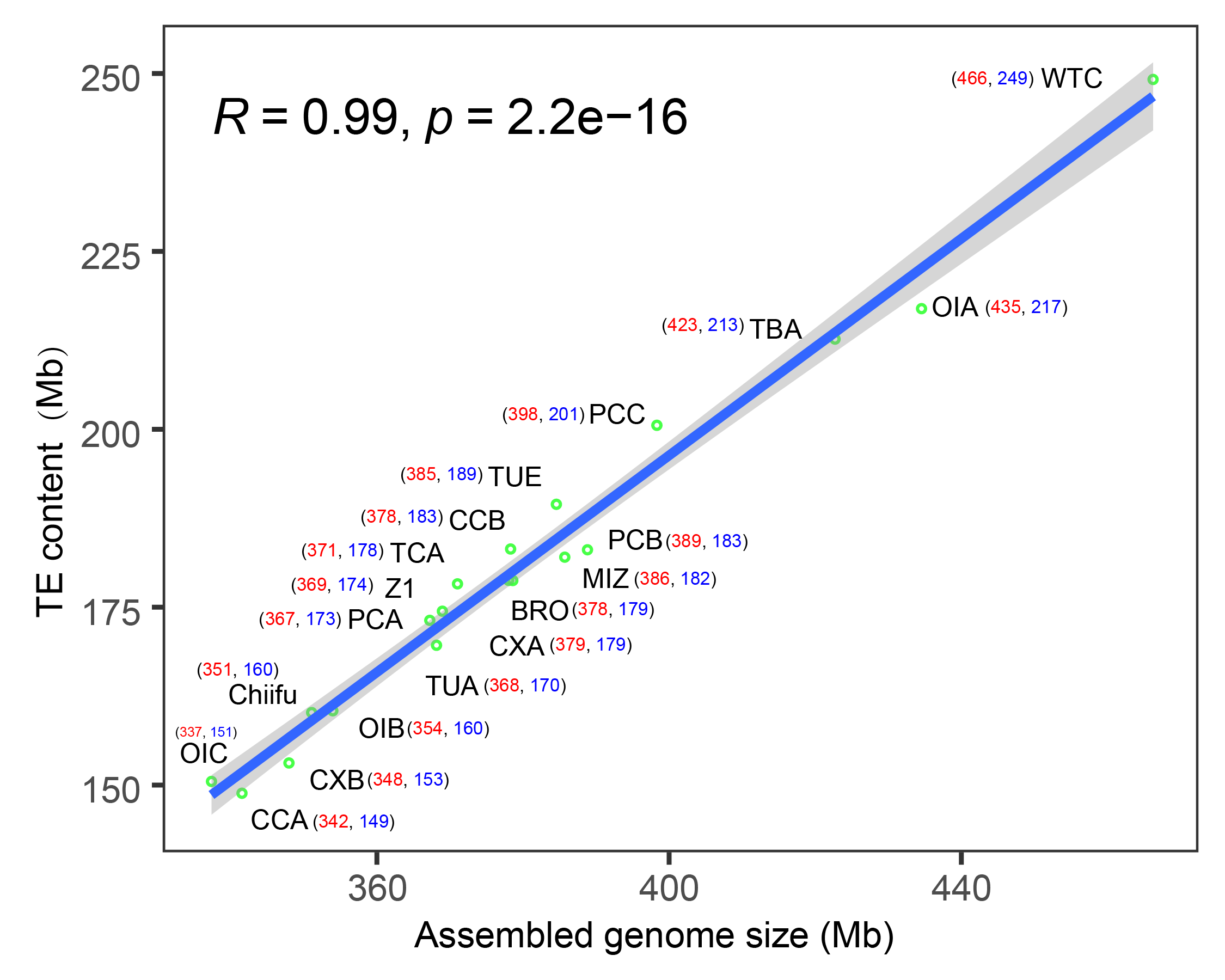
**

**Fig. S2 Correlation analysis between transposable element (TE) content and the assembled genome size of each *B. rapa* accession.** The red and blue numbers represent the assembled size and predicted TE content in each *B. rapa* accession.


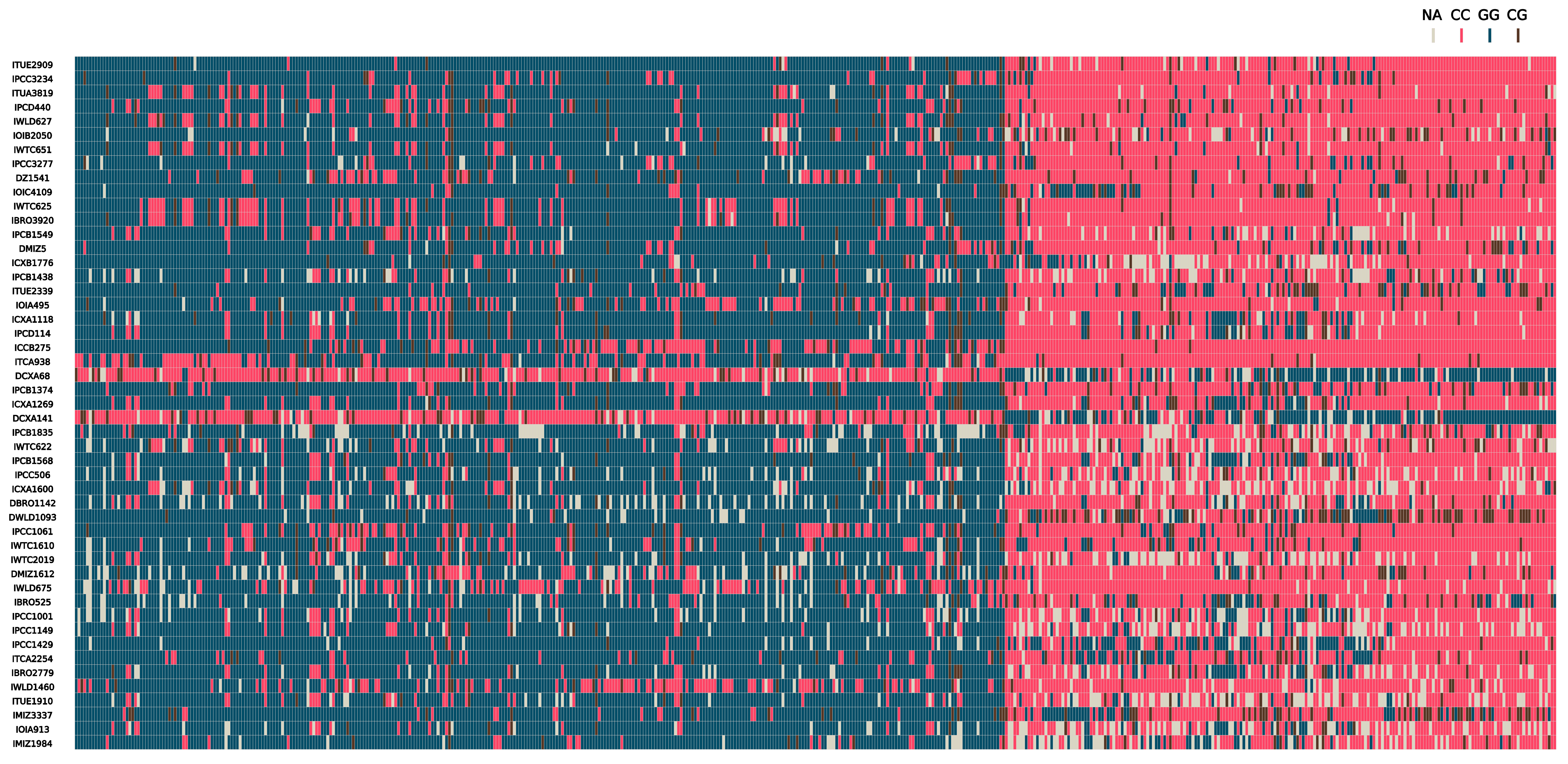


**Fig. S3 The genotypes for the top 50 selection signatures in the heading and non-heading populations in *B. rapa* using transposable element insertion polymorphisms (TIPs) in the unaligned regions.** CC indicates that the genotype in the corresponding accession was consistent with the reference genome, and GG indicates that the genotype in the accession was different from the reference genome, while missing loci (NN) and heterozygous loci (Hetero) are filled with gray and brown.


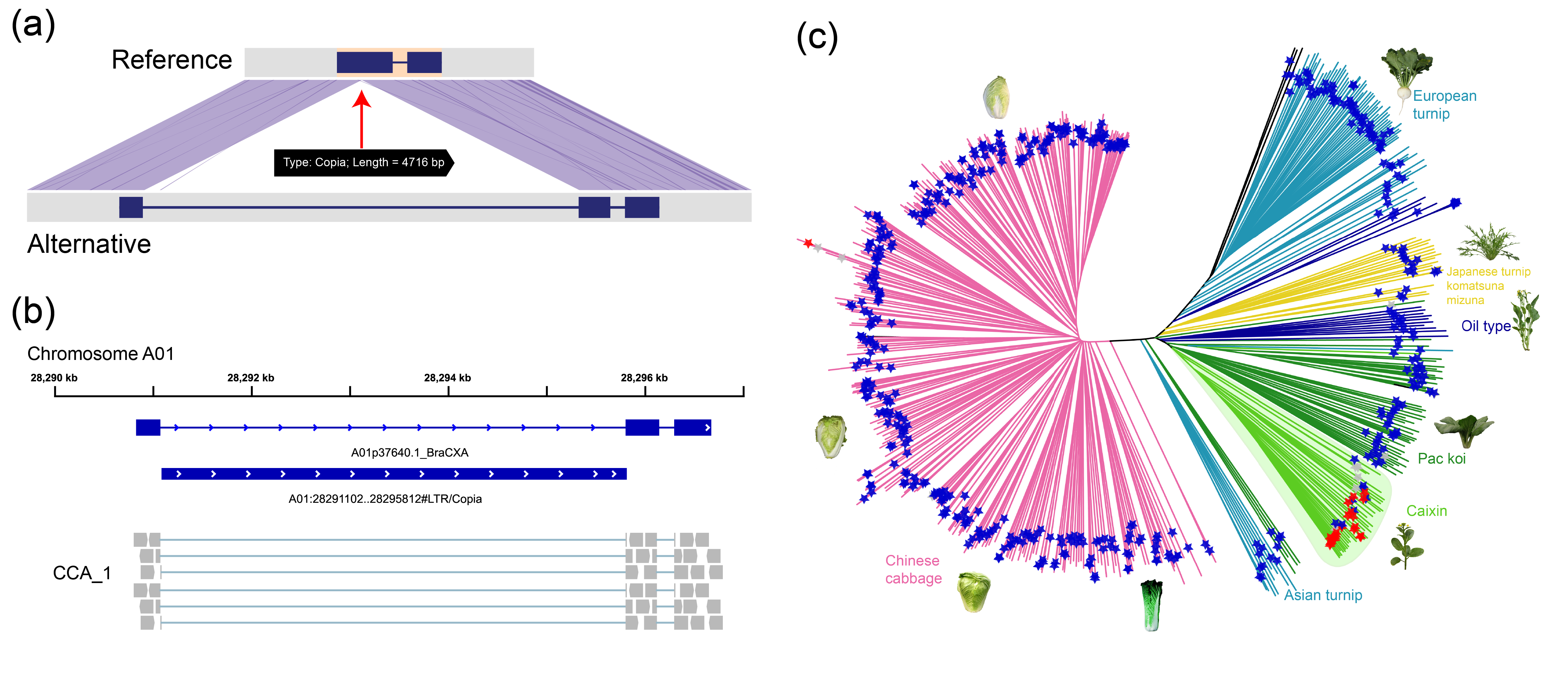


**Fig. S4 A Copia insertion in *BrFLOR1.2* was associated with the domestication of caixin.** (a) An example of a Copia retrotransposable element inserted in coding regions of *BrFLOR1.2* in the caixin genome. (b) An Copia insertion in *BrFLOR1.2* resulted in a new alternative splicing of *BrFLOR1.2*. (c) The landscape of the insertion of the Copia retrotransposable element in 524 *B. rapa* genomes. The red star indicates that the accession harbors the Copia insertion.





**Fig. S5 The distribution of haplotypes in the *BrFLOR1.2* gene region in 524 genomes**. The colors used are the same as in Fig. 7.


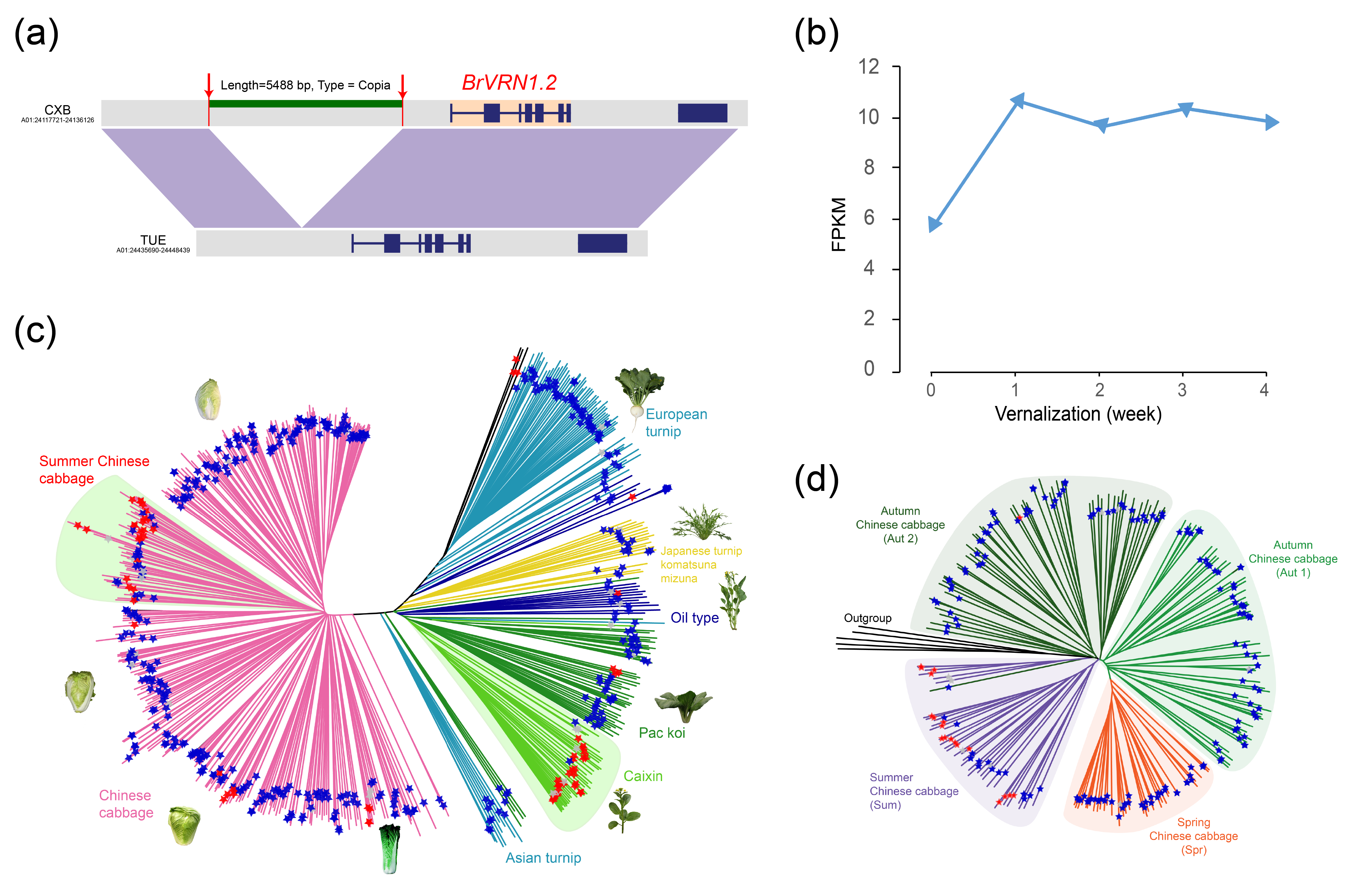


**Fig. S6 A Copia insertion in *BrVRN1.2* was associated with the domestication of caixin and summer Chinese cabbage.** (a) An example of a Copia retrotransposable element inserted in the upstream region of *BrVRN1.2*. (b) The expression level of *BrVRN1.2* under different vernalization. (c) The landscape of the insertion of the Copia retrotransposable element in 524 *B. rapa* genomes. The red star indicates that the accession harbors the Copia insertion.





**Fig. S7 The distribution of haplotypes in the *BrVRN1.2* gene region in 524 genomes.** The colors used are the same as in Figure 7.


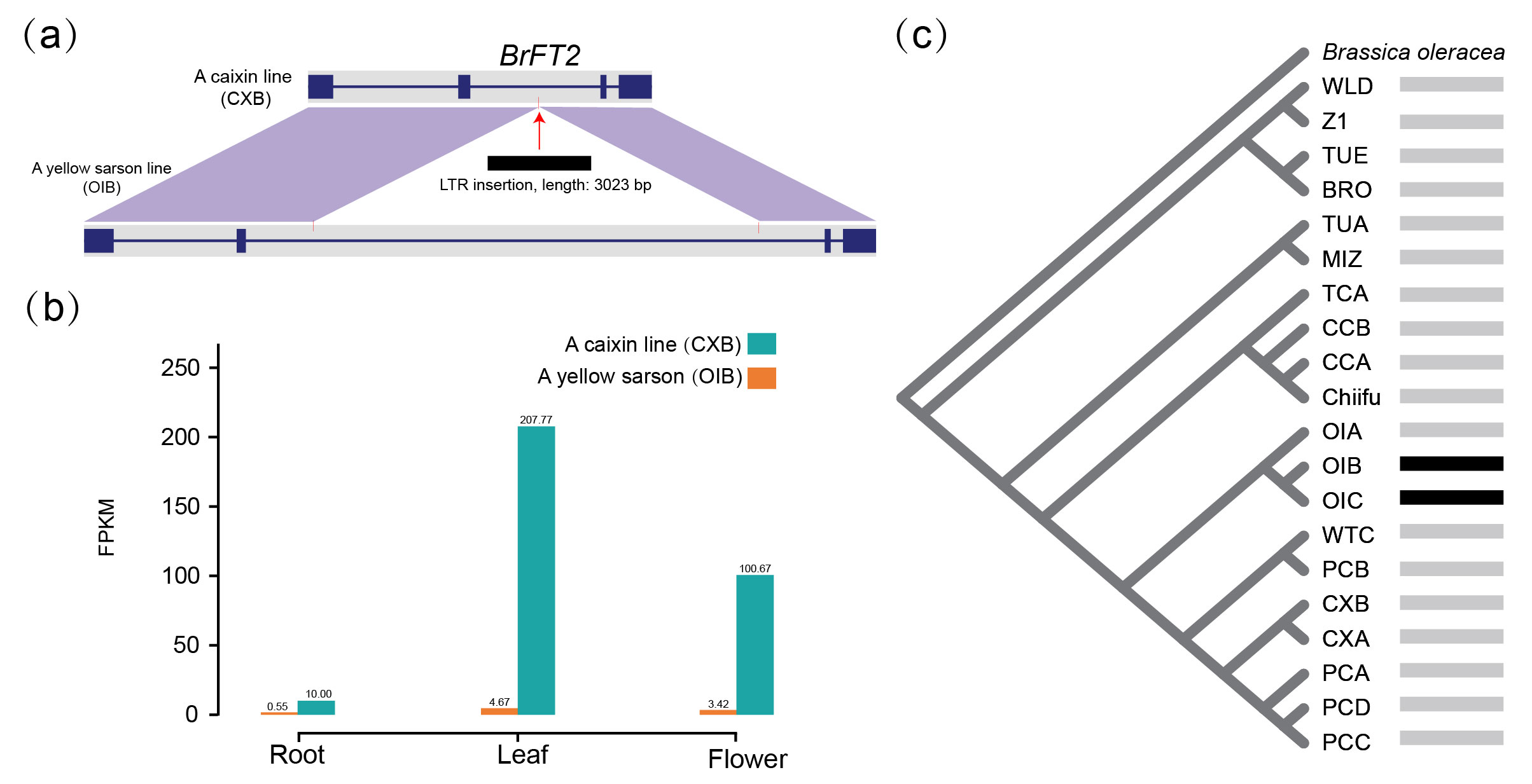


**Fig. S8 An LTR insertion in *BrFT2* gene.** (a) a 3023 bp LTR repeat element inserted in a yellow sarson line (OIB) while being absent in a caixin line (CXB). (b) Expression analysis of the *BrFT2* gene in CXB and OIB. (c) The LTR insertion in 20 *B. rapa* genomes.


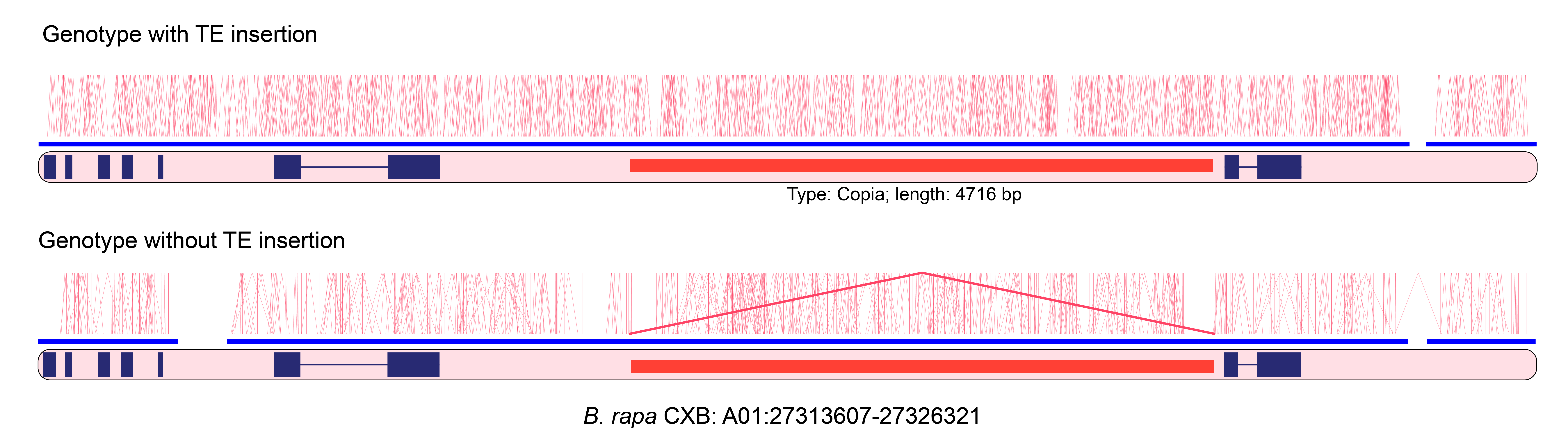


**Fig. S9 The validation of transposable element (TE) insertions among different *B. rapa* genomes based on the insertion sizes of paired-end reads.**
