# Supplementary material for: Transposable element insertion: a hidden major source of domesticated phenotypic variation in Brassica rapa
Source: Plant Biotechnol J. 2022 Mar 18;20(7):1298–310. doi: 10.1111/pbi.13807 (PMC9241368; doi:10.1111/pbi.13807)
Supplement: Supplementary file 3 — Appendix S1 The pipeline to retrieve population‐scale TIPs. [file PBI-20-1298-s002.docx]

**Supplementary Notes**

**The pipeline to retrieve population-scale TIPs**

Identification of insertions and deletions in the *B. rapa* pan-genome. In this step, we used each of the 20 *B. rapa* genomes as the reference and identified insertions and deletions in the pan-genome (Fig. 2A). For example, we identified 107.8 Mb of deletions that were present in the Chiifu-401 genome but absent in at least one of the other 19 genomes. Additionally, we identified 409.1 Mb of insertions that were absent in the Chiifu-401 genome but present in one or more of the other 19 genomes. This process was repeated sequentially for all genomes. In total, we identified 93.2–123.9 Mb deletions and 369.1–418.6 Mb insertions in each of the 20 genomes (Table S2).

Construction of the TE insertion dataset. After obtaining insertions and deletions, we mapped each insertion or deletion onto the *B. rapa* TE library. If the similarity and coverage of one deletion (or insertion) was greater than 80% (also called ‘the 80-80 rule’), then the deletion (or insertion) was defined as a TE insertion. Furthermore, we proposed the concepts of ‘aligned regions’ and ‘unaligned regions’ to describe TIPs in the pan-genome. The concepts were based on Chiifu-401 genomic sequences. If genomic sequences from the other 19 accessions could be covered by the Chiifu-401 sequences, we denoted such regions as being ‘aligned regions’; if the genomic sequences in the other genomes could not be covered by the Chiifu-401 sequences, we defined them as ‘unaligned regions’ (Fig. 2B). With this criterion, we found that the 19 genomes contained 63.64–86.68% aligned regions and 13.32–36.36% unaligned regions (Table S3).

Determination of TIPs at a population scale. We implemented the strategy by mapping the short-read datasets onto the TE insertions and their flanking sequences. If one or two boundaries for a TE insertion were covered by the short reads, we defined this accession as harboring the same TE insertion (see method) (Fig. 2C). The detailed process included three steps: we first extracted the flanking sequences of each TE insertion (including 1 kb upstream and downstream of the TE insertion); then, the upstream flanking sequence, the TE insertion sequence, and the downstream flanking sequence were linked together in order. After that, we mapped the population-scale resequencing short reads onto our constructed target sequences. If a read in one accession was directly aligned to the upstream and downstream flanking sequences, we considered that there was no TE insertion in this accession; if a read in one accession was directly aligned to the TE insertion sequence and at least one flanking sequence (upstream or downstream flanking sequence), then the accession was considered to harbor the same TE insertion.
